# Mechanisms underlying university students' perceived AI threat: the mediating roles of perceived controllability and perceived effort–reward imbalance

**DOI:** 10.3389/fpsyg.2026.1875419

**Published:** 2026-08-25

**Authors:** Yuetong Liang, Juan Du

**Affiliations:** 1 Ontario Institute for Studies in Education, University of Toronto, Toronto, ON, Canada; 2 Faculty of Information Technology, Monash University, Melbourne, VIC, Australia

**Keywords:** AI anxiety, AI self-efficacy, growth mindset, human–AI relationship experience, perceived AI threat, perceived controllability, perceived effort–reward imbalance, role overload

## Abstract

**Introduction:**

As artificial intelligence (AI) becomes increasingly integrated into educational assessment and employment practices, university students are becoming more concerned about their future career opportunities, the value of their skills, and employment competition. However, the mechanisms underlying perceived AI threat remain insufficiently understood among undergraduate and postgraduate students in the humanities and social sciences in China. This study develops an integrated model in which perceived controllability and perceived effort-reward imbalance serve as parallel mediators and examines how human–AI relationship experience, AI anxiety, AI self-efficacy, growth mindset, and role overload influence perceived AI threat.

**Methods:**

This cross-sectional study analyzed 548 valid questionnaires. SPSS 26.0 and SmartPLS 3.0 were used to conduct descriptive analyses, assess the measurement model, estimate the structural model, and perform bootstrapping tests of the mediation effects.

**Results:**

Perceived controllability significantly and negatively predicted perceived AI threat, whereas perceived effort–reward imbalance significantly and positively predicted perceived AI threat. Perceived controllability significantly mediated the relationships between each of the five antecedent variables and perceived AI threat. Perceived effort–reward imbalance significantly mediated the effects of AI self-efficacy, growth mindset, and role overload on perceived AI threat, whereas its mediating effects in the relationships involving AI anxiety and human–AI relationship experience were not statistically significant. Role overload produced the largest total effect on perceived AI threat. Growth mindset had no significant direct effect but produced significant indirect effects through both mediators.

**Discussion:**

University students' threat judgments regarding AI are associated both with whether they believe they can cope with technological change and with whether they believe their current investments can generate commensurate developmental returns. These findings clarify the dual appraisal mechanisms underlying perceived AI threat and provide empirical evidence supporting the implementation of AI literacy education, psychological adaptation support, and career development guidance in universities.

## 1 Introduction

Artificial intelligence is rapidly changing the operation of higher education and the labor market. AI systems have been used for learning support, academic assessment, recruitment screening, performance evaluation, and skills assessment (Crompton and Burke, 2023; Ali and Kallach, 2024). For university students who are still transitioning from education to employment, these changes represent new opportunities for learning and development, but they may also generate concerns about skill devaluation, job displacement, career competition, and fairness in evaluation. Previous research has found that university students' AI anxiety is significantly associated with unemployment anxiety (Uçar et al., 2025), and that the use of AI in organizational settings may undermine individual wellbeing through identity threat and cognitive job insecurity (Gull et al., 2023). Therefore, understanding why and how university students form threat judgments regarding AI has become an important issue in AI education research and student development research.

Although the field of AI research is expanding rapidly (Mustafa et al., 2024; Qin et al., 2024), several important research gaps remain unexplored. First, the literature has long centered on technology acceptance, behavioral intention, and adoption behavior, with particular attention to how performance expectancy, self-efficacy, work stress, or digital competence affects AI use, while giving relatively limited attention to the psychological threats, concerns about skill value, and career uncertainty that AI may generate (Acosta-Enriquez et al., 2025; Rodríguez-Ruiz et al., 2025). Second, although emerging research has begun to examine AI-related anxiety and unemployment concerns among university students (Ucar et al., 2025), as well as AI-induced identity crises and job insecurity in workplace settings (Gull et al., 2023), factors such as AI anxiety, self-efficacy, technology interaction experience, and task demands are often studied separately, and few studies have examined within a single model how these psychological resources and contextual stressors operate jointly. Third, although mechanisms such as perceived controllability and effort–reward imbalance have been widely examined in occupational settings, particularly in studies involving employees facing technological change, such as Gull et al. (2023), research on these mechanisms among university students in AI-related contexts remains limited. Existing research in educational psychology indicates that effort–reward imbalance significantly affects university students' learning engagement (Zhang et al., 2025). However, these studies have not been situated within the context of AI-driven labor market transformation. Notably, most studies of AI-related stress and identity threat are based on employee samples and focus on current job security and organizational roles, whereas university students are in a transitional stage of career preparation, and the mechanisms through which perceived controllability and expectations of future returns operate may differ. Therefore, directly applying employee-based psychological models to student populations may overlook developmental-stage and contextual differences. These mechanisms need to be examined in depth among students to clarify how AI-related threats are psychologically processed during career preparation. Addressing these gaps is therefore essential for developing a more comprehensive framework that simultaneously encompasses the psychological and contextual factors underlying university students' perceptions of AI threat.

Cognitive appraisal theory proposes that external change does not automatically produce a uniform stress response; rather, individuals form different cognitive appraisals according to the importance of an event, its potential consequences, and the resources available to them (Lazarus and Folkman, 1984). In the context of AI, students may first judge whether they can learn about, understand, and adapt to AI-related changes, while also judging whether the time and effort invested in adapting to these changes can be converted into corresponding academic, capability-related, and career returns. The former judgment can be represented by AI-related perceived controllability, whereas the latter can be represented by perceived effort–reward imbalance. The two concepts are distinct: perceived controllability concerns whether external constraints are manageable, whereas perceived effort–reward imbalance concerns whether investments match expected returns.

Based on the above gaps, this study treats human–AI relationship experience, AI anxiety, AI self-efficacy, growth mindset, and role overload as antecedent variables; perceived controllability and perceived effort–reward imbalance as two parallel mediators; and perceived AI threat as the outcome variable, thereby constructing a parallel multiple-mediation model. The model does not assume a causal sequence between the two appraisals but simultaneously examines their respective direct predictive and indirect effects. The study focuses on senior undergraduate and postgraduate students in the humanities and social sciences in China because their disciplinary competence is generally not centered on computing technologies, making uncertainty about the value of their skills and future career returns theoretically relevant in the context of AI's rapid transformation of knowledge production and occupational tasks.

## 2 Literature review and hypothesis development

### 2.1 Perceived AI threat

Perceived AI threat refers to the extent to which individuals subjectively believe that AI technologies may harm their personal opportunities, the value of their capabilities, career development, or social status (Li, 2023). This construct is not equivalent to a generally negative attitude toward AI. A general negative attitude may encompass broad doubts about ethics, fairness, reliability, or technological errors, whereas threat perception places greater emphasis on the connection between the consequences of AI and individuals' own interests, such as concerns about reduced employment opportunities, declining career competitiveness, the devaluation of existing knowledge, or diminished autonomy. University students' threat judgments are clearly future-oriented. On the one hand, automation and machine learning are changing job structures and skill requirements, and students may worry that their current educational backgrounds will be insufficient for the future labor market (Tiwari, 2023). On the other hand, AI is increasingly involved in learning assessment, talent screening, and important decision-making. When students regard these systems as difficult to understand, predict, or influence, they may experience reduced autonomy and a loss of control over outcomes (Jones-Jang and Park, 2023). In addition, the wage premium associated with AI-related skills may intensify students' concerns about unequal competition, making those who lack such capabilities more likely to interpret technological development as a structural risk (Grant and Üngör, 2024). Perceived AI threat is not determined solely by technological characteristics. Individuals' AI knowledge, frequency of use, emotional responses, capability beliefs, and current stress levels may all influence their judgments (Majerek et al., 2025). Accordingly, this study conceptualizes perceived AI threat as a subjective appraisal outcome jointly shaped by individual resources, interaction experience, and contextual demands.

Previous research has found that AI anxiety is closely associated with individuals' perceptions of AI-related risks, uncertainty, and negative outcomes. For example, Chen et al. (2025) found that AI anxiety increases individuals' perceptions of uncertainty when they encounter AI-related situations, whereas higher self-efficacy can reduce this negative psychological response. In addition, research on student populations has shown that fear and threat perceptions related to generative AI further influence students' attitudes toward and behavioral intentions to use AI (Yin, 2025). These studies indicate that how students evaluate their ability to respond to AI-related change is an important factor in understanding the formation of perceived AI threat.

### 2.2 Theoretical foundation and the two mediating variables

Cognitive appraisal theory states that, when facing a potentially stressful event, individuals assess whether the event is relevant to their goals and wellbeing and determine whether they possess sufficient resources to cope with it (Lazarus and Folkman, 1984). The same technological change may therefore be interpreted by different students as an opportunity, a challenge, or a threat. Students with more AI-use experience, stronger capability beliefs, and more sufficient time resources may regard AI-related change as learnable, usable, and manageable. By contrast, students with higher anxiety or prolonged task overload may be more likely to focus on displacement risks and uncertain consequences. Using cognitive appraisal theory as the overarching explanatory perspective, this study treats perceived controllability and perceived effort–reward imbalance as two mediating cognitive judgments that can occur in parallel, without assuming a causal sequence between them. Perceived controllability reflects students' judgments of external constraints and room for adaptation, whereas perceived effort–reward imbalance reflects their judgments of the extent to which investments match expected returns.

Perceived controllability refers to the extent to which individuals believe that they can understand, influence, manage, or adapt to specific environmental changes and their potential consequences. Research on perceived control indicates that, when individuals believe environmental changes can be adjusted to some extent through their own actions, they generally exhibit more positive adaptive responses; when they believe that events are developing beyond the scope of their abilities and resources, they are more likely to experience helplessness, stress, and threat appraisals (Skinner, 1996). The sense-of-control scale developed by Lachman and Weaver (1998a) and Lachman and Weaver (1998b) distinguishes personal mastery from perceived constraints, with perceived constraints focusing on whether individuals believe that the external environment impedes goal attainment. This study examines university students' perceived controllability on the basis of the perceived constraints dimension and is therefore distinct from AI self-efficacy.

Effort–reward imbalance theory conceptualizes stress as a state of non-reciprocity between high investment and low reward (Siegrist, 1996). Previous research has shown that effort–reward imbalance is closely associated with negative psychological outcomes in educational settings. For example, Liu et al. (2024) found that effort–reward imbalance reduces university students' learning engagement through learned helplessness, and Tang and Wang (2026) further reported a positive association between effort–reward imbalance and students' academic anxiety. In the context of AI, when students believe that they need to invest substantial time in learning AI-related skills but that their future career returns remain highly uncertain, they may be more likely to believe that AI will weaken their competitive advantage, thereby increasing perceived AI threat. Effort–reward imbalance concerns an individual's appraisal of the degree of fit between investments and expected returns, namely, “Will the effort I expend yield corresponding rewards?” In the context of AI, students may not necessarily believe that they will be unable to obtain employment, nor may they directly fear that occupations will disappear; rather, they may be concerned about whether the substantial time invested in learning new skills to adapt to the AI era can be translated into future career advantages. Therefore, effort–reward imbalance emphasizes judgments about the value of investment and the fairness of returns, whereas employability concerns and job insecurity emphasize future employment outcomes themselves. Traditional ERI questionnaires generally measure effort, reward, and overcommitment separately and may further be used to calculate an imbalance ratio. This study did not directly adopt the dimensional structure or ratio of the traditional ERI questionnaire. Instead, based on the theory's core definition of a mismatch between investment and return, five context-specific items were developed to measure students' overall subjective appraisal of the mismatch between AI-related learning investments and expected academic, capability-related, and career returns. This construct was named perceived effort–reward imbalance (ERI). It demonstrated satisfactory preliminary psychometric properties in the present sample.

### 2.3 Antecedents of perceived controllability

Previous research has shown that individuals' experiences of interacting with artificial intelligence systems influence their perceptions of AI, their trust in AI, and their subsequent behavioral responses. Positive human–AI interaction experiences can increase users' familiarity with and trust in AI systems, help them develop a more accurate understanding of AI's capability boundaries and operating mechanisms, and thereby reduce uncertainty regarding AI-related changes (Hoff and Bashir, 2015; Glikson and Woolley, 2020). When students have more positive experiences interacting with AI tools in learning and daily life, they are more likely to perceive AI-related changes as understandable, controllable, and adaptable, thereby strengthening their sense of control over the development of AI. Accordingly, this study proposes:

H1a: Human–AI relationship experience positively affects perceived controllability.

AI anxiety refers to individuals' concerns about uncertainty in the field of artificial intelligence and whether their own skills are sufficient to meet emerging demands (Chen et al., 2025). Research has shown that anxiety regarding AI and related technologies weakens individuals' perceptions of their own capacity for control, thereby contributing to negative attitudes and resistance (Verma et al., 2025). For example, research in higher education has demonstrated that AI anxiety significantly inhibits individuals' attitudes toward AI applications (Tailor and Tailor, 2025). Accordingly, this study proposes:

H2a: AI anxiety negatively affects perceived controllability.

Self-efficacy refers to individuals' beliefs in their ability to perform the actions required to achieve specific goals or complete particular tasks (Egele et al., 2025); in the present context, it reflects confidence in one's ability to use and manage AI tools. Empirical research has shown that higher AI self-efficacy strengthens users' positive perceptions of their own capabilities and reduces anxiety, thereby enhancing their sense of control in technological environments (Chen et al., 2025). Accordingly, this study proposes:

H3a: AI self-efficacy positively affects perceived controllability.

Growth mindset refers to the belief that abilities, skills, and intelligence can be improved through sustained effort, learning, and practice (Dweck, 2006). According to cognitive appraisal theory, individuals' interpretations of environmental changes depend not only on the external events themselves but also on their perceptions of their own developmental potential and coping resources. Students with a growth mindset are more likely to view AI-induced changes in skill requirements as a process to which they can adapt through learning and effort rather than as an immutable threat. Therefore, a growth mindset may strengthen students' sense of control over AI-related changes. Accordingly, this study proposes:

H4a: Growth mindset positively affects perceived controllability.

Role overload refers to a state in which the tasks and responsibilities undertaken by an individual exceed the time, capabilities, or psychological resources available to that individual (Peterson et al., 1995; Tang and Vandenberghe, 2021). According to cognitive appraisal theory, when individuals face demands that exceed their available resources, their sense of control over the environment is weakened, and they are more likely to perceive external changes as threatening. In the context of rapid AI development, students must not only complete traditional learning tasks but also continually acquire new digital skills and AI-related capabilities. When students perceive that their available resources are insufficient to meet these additional demands, their sense of control over AI-related changes may decline. Accordingly, this study proposes:

H5a: Role overload negatively affects perceived controllability.

### 2.4 Antecedents of perceived effort–reward imbalance

Positive human–AI interaction experiences may influence not only students' judgments of their ability to manage AI-related changes but also their evaluations of the value of AI-related investments (Glikson and Woolley, 2020). When students believe that AI tools can effectively support learning, information acquisition, and personal development, they are more likely to believe that investing time and effort in learning about and adapting to AI technologies will yield corresponding returns rather than result in an imbalanced state of “high effort and low reward.” Therefore, positive human–AI interaction experiences may alleviate students' perceived effort–reward imbalance. Accordingly, this study proposes:

H1b: Human–AI relationship experience negatively affects perceived effort–reward imbalance.

AI anxiety affects not only individuals' evaluations of their own coping capabilities but also their perceptions of the value of investment in the AI era (Wang and Wang, 2022). When students are concerned that their skills may be unable to keep pace with AI development or believe that their existing knowledge and capabilities may rapidly depreciate, they may be more likely to question whether investing time and effort in learning AI-related skills will yield corresponding returns, thereby increasing perceived effort–reward imbalance. Accordingly, this study proposes:

H2b: AI anxiety positively affects perceived effort–reward imbalance.

AI self-efficacy may also influence students' judgments of the value of AI-related investments (Wang and Chuang, 2024). When students believe that they can effectively learn and use AI tools, they are more likely to believe that investing time and effort in developing AI-related capabilities will generate future returns rather than result in an imbalance characterized by substantial effort and limited reward. Therefore, higher AI self-efficacy may alleviate perceived effort–reward imbalance. Accordingly, this study proposes:

H3b: AI self-efficacy negatively affects perceived effort–reward imbalance.

Growth mindset may also influence students' perceptions of the relationship between investment and future returns. Research has shown that growth mindset can mitigate the negative effects of academic stress (Zhang et al., 2025). Therefore, students with a growth mindset are more likely to believe that investing time and effort in learning new skills will generate future returns, thereby reducing their perceived effort–reward imbalance. Accordingly, this study proposes:

H4b: Growth mindset negatively affects perceived effort–reward imbalance.

Role overload may also influence students' perceptions of the relationship between effort and reward. Effort–reward imbalance theory proposes that individuals experience imbalance and stress responses when they expend substantial effort but fail to receive corresponding rewards (Siegrist, 1996). Research in educational psychology has shown that high workload can lead to negative psychological outcomes such as stress and burnout, which is consistent with ERI theory (Ma et al., 2026). For university students, excessive learning demands and pressure to develop new skills may lead them to believe that substantial investments of time and effort are difficult to convert into future competitive advantages, thereby increasing perceived effort–reward imbalance. Accordingly, this study proposes:

H5b: Role overload positively affects perceived effort–reward imbalance.

### 2.5 Direct predictors of perceived AI threat

Previous research on trust in technology has shown that familiarity with technology and positive experiences of technology use can reduce technology-related uncertainty and risk perceptions (Hoff and Bashir, 2015). In the context of AI, individuals who lack relevant knowledge and experience are more likely to form negative evaluations because of unfamiliarity, whereas those with extensive and positive AI-use experience are more likely to view AI as a useful tool rather than a potential threat. Therefore, human–AI relationship experience may reduce students' perceived threat associated with AI development. Accordingly, this study proposes:

H1c: Human–AI relationship experience negatively affects perceived AI threat.

Previous research has shown that technology anxiety increases individuals' perceptions of technological risks, uncertainty, and potential negative consequences (Agarwal and Karahanna, 2000). In the context of AI, individuals with higher levels of anxiety are more likely to focus on the risks of unemployment, skill displacement, and broader social consequences associated with AI and to interpret AI development as a potential threat. Therefore, AI anxiety may intensify students' concerns about AI. Accordingly, this study proposes:

H2c: AI anxiety positively affects perceived AI threat.

Self-efficacy can reduce the uncertainty and threat perceptions that individuals experience when encountering new technologies. Previous research on technology acceptance has shown that individuals with higher levels of technology self-efficacy are more likely to proactively address technological challenges and to regard technology as a usable resource rather than a potential risk (Compeau and Higgins, 1995). In the context of AI, students with higher AI self-efficacy are more likely to believe that AI can be learned and controlled and are therefore less likely to perceive it as a threat. Accordingly, this study proposes:

H3c: AI self-efficacy negatively affects perceived AI threat.

Growth mindset can help individuals reduce threat perceptions when facing uncertain environments and technological change. Previous research has shown that individuals with a growth mindset are more likely to view challenges as opportunities for learning and development rather than as signals that their abilities are under threat (Yeager and Dweck, 2020). Therefore, students with a growth mindset may be more likely to regard AI as a learnable and adaptable tool rather than a potential threat. Accordingly, this study proposes:

H4c: Growth mindset negatively affects perceived AI threat.

Role overload may also influence students' appraisal of the relationship between effort and reward. Effort-reward imbalance theory proposes that when individuals expend substantial effort but cannot obtain commensurate rewards, they experience imbalance and stress responses (Siegrist, 1996). Research in educational psychology has shown that high workload is associated with negative psychological consequences such as stress and burnout, which is consistent with ERI theory (Ma et al., 2026). For university students, excessive learning demands and pressure to develop skills may lead them to believe that substantial investments of time and effort are difficult to translate into future competitive advantages, thereby increasing perceived effort-reward imbalance. Accordingly, this study proposes:

H5c: Role overload positively affects perceived AI threat.

Perceived controllability refers to the extent to which individuals believe that they can influence, manage, or control a particular situation and its potential outcomes (Leotti and Delgado, 2011). According to cognitive appraisal theory, individuals do not immediately respond negatively when confronted with a potential threat. Instead, they first assess whether they possess sufficient capabilities and resources to cope with the situation. When individuals believe that they can effectively control and adapt to environmental changes, they are more likely to view uncertainty as a manageable challenge rather than an unavoidable threat. In technology-use contexts, previous research has shown that higher perceived controllability reduces individuals' perceptions of technological risk and uncertainty and promotes more positive attitudes toward technology (Jones-Jang and Park, 2023). Therefore, in the context of rapid AI development, students with higher perceived controllability may believe that they can adapt to AI-related changes through learning and capability development, thereby reducing their concerns about the potential negative effects of AI. Accordingly, this study proposes:

H6: Perceived controllability negatively affects perceived AI threat.

Effort–reward imbalance theory proposes that when individuals perceive a mismatch between the effort they expend and the rewards they receive, they experience stress responses and negative psychological states (Siegrist, 1996). Although this theory was originally applied primarily to occupational stress research, recent studies have shown that it is also applicable to educational settings and can explain students' stress experiences and adaptation difficulties in highly demanding environments. In the context of rapid AI development, university students must not only invest time in completing their academic tasks but also continually acquire new digital skills and AI-related capabilities. When students believe that their efforts are difficult to convert into corresponding academic progress, employment opportunities, or future career advantages, they may experience stronger perceived effort–reward imbalance and become more concerned about the competitive pressure and career uncertainty associated with AI. Previous research has demonstrated that effort–reward imbalance is significantly associated with poor learning outcomes and psychological stress among university students (Zhang et al., 2025). Therefore, this study conceptualizes perceived effort–reward imbalance as a psychological mechanism based on the appraisal of future outcomes that may strengthen students' perceptions of AI development as threatening. Accordingly, this study proposes:

H7: Perceived effort–reward imbalance positively affects perceived AI threat.

### 2.6 The mediating roles of perceived controllability and perceived effort–reward imbalance

According to cognitive appraisal theory, individuals' threat judgments regarding external change are not directly determined by the event itself but depend on how they evaluate the event's importance, potential impact, and the coping resources available to them (Lazarus and Folkman, 1984). When facing rapidly developing AI technologies, students do not automatically regard AI development as a threat; rather, they make cognitive appraisals based on their own capabilities, sense of control, and expectations of future outcomes. Therefore, psychological and contextual factors that influence perceived AI threat may not act directly on threat judgments but may indirectly shape individuals' understanding of AI-related threats through different appraisal mechanisms.

First, perceived controllability reflects the extent to which individuals believe that they can influence, manage, and adapt to AI-related change. According to self-efficacy theory, positive appraisals of one's own capabilities and control resources enhance adaptability in uncertain environments and reduce threat responses in stressful situations (Bandura, 1997). Therefore, students with more positive human–AI interaction experiences, higher AI self-efficacy, or a stronger growth mindset may develop a stronger sense of control, thereby reducing their threat appraisals of AI development. By contrast, AI anxiety may weaken individuals' positive appraisals of their own capabilities, whereas role overload may deplete available resources and further reduce their sense of control. This study therefore proposes that perceived controllability mediates the relationships between these factors and perceived AI threat.

Second, perceived effort–reward imbalance reflects individuals' appraisal of whether their investments will yield corresponding future returns. Effort–reward imbalance theory proposes that when individuals perceive a mismatch between the effort they expend and the rewards they receive, they experience stress responses that further affect their psychological health and behavioral outcomes (Siegrist, 1996). In the context of AI, human–AI interaction experience, AI self-efficacy, and growth mindset may help students form more positive effort–reward appraisals, whereas AI anxiety and role overload may increase concerns about insufficient future returns. Perceived effort–reward imbalance may therefore further explain how different factors influence perceived AI threat.

Accordingly, this study proposes:

H8: Perceived controllability mediates the relationships between antecedent variables (AI anxiety, AI self-efficacy, human–AI relationship experience, growth mindset, and role overload) and perceived AI threat.

H9: Perceived effort–reward imbalance mediates the relationships between antecedent variables (AI anxiety, AI self-efficacy, human–AI relationship experience, growth mindset, and role overload) and perceived AI threat.

### 2.7 Research model

**Figure 1:**
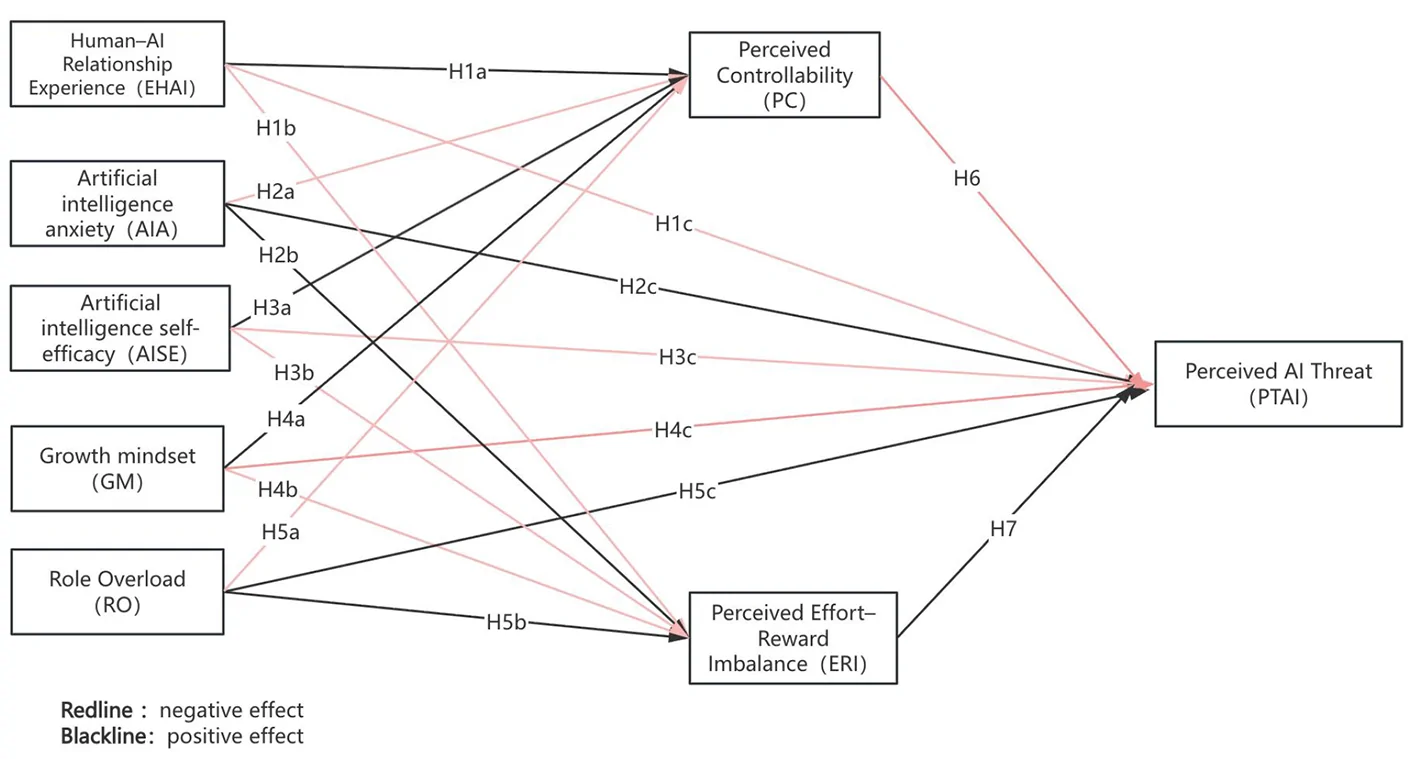
Conceptual framework.

Based on the hypotheses of this study, the structural model treats human–AI relationship experience, AI anxiety, AI self-efficacy, growth mindset, and role overload as independent variables; perceived controllability and perceived effort–reward imbalance as mediating variables; and perceived AI threat as the dependent variable (See Figure 1).

## 3 Research method

### 3.1 Participants and research procedure

The participants were undergraduate and postgraduate students majoring in the humanities and social sciences at higher education institutions in different regions of China. Data were collected from February to March 2026. A combination of convenience sampling and snowball sampling was used to recruit participants. The research team first distributed survey invitations through university teachers, student organizations, and university student social networks and asked eligible participants to forward the questionnaire information to other university students with similar disciplinary backgrounds. The participating institutions included universities under China's Double First-Class initiative, former Project 985 and Project 211 universities, key undergraduate institutions, and other regular undergraduate institutions located in regions and cities with different levels of economic development. No financial compensation, course credit, or other form of reward was provided for participation in this study.

The inclusion criteria were as follows: (1) formally registered undergraduate or postgraduate students at Chinese higher education institutions; (2) enrollment in a humanities- or social-science-related major; (3) prior exposure to AI technologies or awareness of their applications in learning; and (4) voluntary participation with informed consent. A total of 550 questionnaires were collected. The questionnaire included attention-check items and restricted repeated submissions from the same device or IP address. After data screening, one response was excluded because the core scales were incomplete, one because the respondent did not meet the identity or major criteria, yielding a final sample of 548 complete and valid questionnaires. Because open online recruitment was used, the number of individuals who actually received an invitation could not be accurately determined, and a conventional response rate was therefore not calculated.

This study deliberately focused on students majoring in the humanities and social sciences but did not assume that this group necessarily experiences higher perceived threat than students in other disciplines. The theoretical rationale for selecting this group was that their disciplinary training is generally less centered on computing and engineering skills, and, as AI changes knowledge production, content generation, managerial decision-making, and occupational tasks, their judgments regarding the value of existing skills and future career returns may be particularly salient. Therefore, the findings are primarily applicable to humanities and social sciences students in the Chinese higher education system and should not be generalized directly to all university students without further examination.

Data were collected anonymously through the Wenjuanxing online platform. This study was a non-interventional social science survey and did not collect identifiable information such as names, student identification numbers, or national identification numbers. In accordance with the relevant requirements of the location and institution in which the study was conducted, separate formal ethical approval was not required. Before responding, all participants read the study information and provided informed consent. The information covered the purpose of the study, voluntary participation, the right to withdraw, data confidentiality measures, and the researchers' contact information. The final sample included 11 participants under 18 years of age. The questionnaire required them to confirm that their parents or legal guardians were aware of and had agreed to their participation, and the participants themselves also expressed voluntary consent.

Table 1 presents the demographic characteristics of the participants. Of the 548 participants, 54.6% were male and 45.4% were female. Most participants were fourth-year undergraduates (58.8%), followed by third-year undergraduates (37.4%). Management students constituted the largest disciplinary group (22.6%), followed by economics students (19.0%) and education students (15.1%). Most participants were aged 18–25 years (89.8%). Regarding institution type, 50.2% were enrolled at regular undergraduate institutions, while 25.5% attended Double First-Class universities.

**Table 1:** Demographic characteristics of the participants (*N* = 548).

Characteristic	Category	*N*	%
Gender	Male	299	54.6
	Female	249	45.4
Grade	Third-year undergraduate	205	37.4
	Fourth-year undergraduate	322	58.8
	Master's year 2	14	2.6
	Master's year 3	2	0.4
	Other	5	0.9
Major	Literature	68	12.4
	History	49	8.9
	Philosophy	39	7.1
	Law	65	11.9
	Economics	104	19
	Management	124	22.6
	Education	83	15.1
	Arts	6	1.1
	Other	10	1.8
Age	Under 18	17	3.1
	18–25	492	89.8
	26–30	37	6.8
	31–40	2	0.4
University type	Project 985	25	4.6
	Project 211	53	9.7
	Double first-class	140	25.5
	Regular undergraduate	275	50.2
	Vocational/technical college	55	10

### 3.2 Measurement instruments

The first section of the questionnaire collected demographic information, and the second section contained 57 items measuring eight constructs: AI anxiety (6 items), perceived controllability (10 items), AI self-efficacy (7 items), growth mindset (7 items), role overload (7 items), perceived effort–reward imbalance (5 items), perceived AI threat (5 items), and human–AI relationship experience (10 items). All items were rated on a 7-point Likert scale ranging from 1 (strongly disagree) to 7 (strongly agree). Negatively worded constraint items were reverse-scored before analysis so that higher scores for each construct indicated a higher level of the corresponding characteristic. The measurement instruments were handled in three ways. First, the scales for AI anxiety, AI self-efficacy, growth mindset, human–AI relationship experience, and perceived AI threat were shortened and contextually adapted, retaining the items most relevant to the core definitions of the constructs and the university student context. Second, perceived controllability was based on the perceived constraints dimension of Lachman and Weaver (1998a) and Lachman and Weaver (1998b), with the original eight items retained and two context-specific items added; role overload retained the five items from the role overload dimension of Peterson et al. (1995) and added two items. Third, perceived effort–reward imbalance was not a directly shortened version of the traditional ERI questionnaire but a newly developed five-item unidimensional scale based on the core theoretical definition proposed by Siegrist (1996), the measurement tool has gained preliminary psychometric support in the current sample. The sources, measurement content, and adaptation procedures for all instruments are detailed in Appendix 1. This study further examined the outer loadings of all retained items to assess the measurement quality of the adapted instruments. As shown in Table 2, the outer loadings of all items exceeded the recommended threshold of 0.70 and ranged from 0.750 to 0.877, indicating that all retained indicators had good item reliability and effectively reflected their corresponding latent variables. Therefore, no items were removed because of insufficient outer loadings. The complete questionnaire items are provided in Supplementary material A.

**Table 2:** Outer loadings of the retained measurement items.

Construct	Items	Outer loadings
AI anxiety	AIA1–AIA6	0.835–0.872
AI self-efficacy	AISE1–AISE7	0.782–0.873
Human–AI relationship experience	EHAI1–EHAI10	0.782–0.868
Perceived effort–reward imbalance	ERI1–ERI5	0.818–0.850
Growth mindset	GM1–GM7	0.750–0.856
Perceived controllability	PC1–PC10	0.806–0.877
Perceived AI threat	PTAI1–PTAI5	0.785–0.838
Role overload	RO1–RO7	0.757–0.820

For scales originally developed in English, the research team used a translation–back-translation procedure. First, a bilingual researcher with a background in education and psychology translated the scales into Chinese. A second bilingual researcher who had not seen the original text then translated the Chinese version back into English. The research team compared the original and back-translated versions and discussed and revised semantic discrepancies. Subsequently, two researchers with backgrounds in educational technology, AI in education, or psychometrics assessed the consistency of the items with the construct definitions, their applicability to AI contexts in higher education, and the clarity of their wording, and the final revisions were made accordingly. All constructs were treated as reflective constructs in the structural model. It should be noted that the measurement performance of shortened, extended, and newly developed scales must be re-examined in the current sample and cannot be inferred solely from the established reliability and validity of the original scales. Table 3 lists the source, number of items, and representative item for each construct; full details of the adaptations are provided in Appendix 1.

**Table 3:** Overview of the measurement instruments.

Construct	Source	Number of items	Representative item
AI Anxiety (AIA)	Yang and Sundar (2025), shortened and contextually adapted	6	I feel nervous when I need to use artificial intelligence tools.
Perceived controllability (PC)	Lachman and Weaver (1998a) and Lachman and Weaver (1998b), perceived constraints dimension selected and extended	10	Many AI-related changes affecting my learning and future career are beyond my control.
AI self-efficacy (AISE)	Hong (2022), shortened and contextually adapted	7	Even without direct guidance, I am confident that I can use artificial intelligence tools to complete learning tasks.
Growth mindset (GM)	Sigmundsson and Haga (2024), shortened	7	I believe that I can continuously improve my abilities through effort and learning.
Role overload (RO)	Peterson et al. (1995), role overload dimension selected and extended	7	I have more learning and development tasks than I can reasonably manage.
Perceived effort–reward imbalance (ERI)	Contextually developed for this study based on Siegrist (1996) theory, the measurement tool has gained preliminary psychometric support in the current sample.	5	The effort I invest in adapting to artificial intelligence is not matched by the academic or career benefits I expect to receive.
Perceived AI threat (PTAI)	Schepman and Rodway (2020), with reference to the structural evidence reported by Ţîru et al. (2025)	5	Artificial intelligence may reduce my future employment and career development opportunities.
Human–AI relationship experience (EHAI)	Christoforakos et al. (2021), shortened and contextually adapted	10	The artificial intelligence system I frequently use usually understands what I am trying to express.

### 3.3 Data analysis

SPSS 26.0 and SmartPLS 3.0 were used in stages for the analyses. First, demographic characteristics, means, standard deviations, skewness, and kurtosis were calculated, and common method bias was assessed. Partial least squares structural equation modeling (PLS-SEM) was then used to evaluate the measurement and structural models. PLS-SEM was selected mainly because the study simultaneously included multiple antecedent variables, two mediators, and direct effects, and because the analysis focused on explaining variance in endogenous variables, estimating complex path relationships, and testing specific indirect effects rather than on overall model-fit validation centered on reproducing a covariance matrix. The measurement model was assessed for internal consistency and convergent validity using Cronbach's α, rho_A, composite reliability (rho_C), and average variance extracted (AVE), and discriminant validity was assessed using the heterotrait–monotrait ratio (HTMT). Structural model assessment included inner VIF, effect size *f* ^2^, coefficient of determination *R*^2^, and Stone–Geisser *Q*^2^. Path coefficients and mediation effects were tested using 5,000 bootstrap resamples and two-tailed tests, with standardized path coefficients (β), standard errors, *t*-values and *p*-values. A mediation effect was considered significant when the 95% confidence interval for the indirect effect did not include zero. Questionnaires with missing core measurement data were excluded by listwise deletion before the formal analyses. The final sample of 548 complete responses was substantially larger than the minimum required for model estimation. To assess the robustness of the results, female status, fourth-year undergraduate status, management major, and regular undergraduate institution status were dummy-coded as control variables and specified as predictors of perceived AI threat, and the core paths and explained variance were compared before and after the controls were included.

## 4 Results

### 4.1 Descriptive analysis

As shown in Table 4, the means of the eight constructs ranged from 3.42 to 4.28. AI anxiety (*M* = 4.28, SD = 1.71) and AI self-efficacy (*M* = 4.23, SD = 1.68) were relatively high, whereas perceived effort–reward imbalance (*M* = 3.42, SD = 1.63) and perceived AI threat (*M* = 3.55, SD = 1.58) were close to the scale midpoint. Skewness ranged from −0.12 to 0.31, and kurtosis ranged from −1.27 to −0.92, indicating no severe univariate non-normality. To reduce common method bias, the questionnaire was administered anonymously, and participants were explicitly informed that there were no right or wrong answers. Harman's single-factor test extracted eight factors with eigenvalues greater than 1, and the first factor explained 22.62% of the total variance. Full collinearity VIF values ranged from 1.230 to 1.857, all below 3.3 (Kock, 2015). Therefore, common method bias was unlikely to have seriously affected the study's conclusions.

**Table 4:** descriptive statistics.

Construct	Mean	Standard deviation	Skewness	Kurtosis
AIA	4.278	1.711	−0.119	−1.083
PC	4.162	1.711	−0.121	−1.265
AISE	4.228	1.683	−0.065	−1.144
PTAI	3.545	1.584	0.192	−0.983
GM	4.007	1.586	0.043	−0.962
RO	3.849	1.603	0.145	−0.972
ERI	3.419	1.626	0.305	−0.919
EHAI	4.082	1.653	−0.021	−1.04

### 4.2 Measurement model assessment

As shown in Table 5, Cronbach's α values ranged from 0.861 to 0.955, rho_A values ranged from 0.862 to 0.956, and rho_C values ranged from 0.900 to 0.961, all exceeding 0.70. AVE values ranged from 0.630 to 0.737, all exceeding 0.50, indicating good internal consistency and convergent validity for all reflective constructs.

**Table 5:** Construct reliability and validity.

Construct	Cronbach's alpha	Composite reliability (rho_a)	Composite reliability (rho_c)	Average variance extracted (AVE)
**AIA**	0.929	0.935	0.944	0.737
**AISE**	0.930	0.935	0.943	0.704
**EHAI**	0.951	0.954	0.958	0.693
**ERI**	0.893	0.894	0.921	0.700
**GM**	0.902	0.906	0.923	0.630
**PC**	0.955	0.956	0.961	0.712
**PTAI**	0.861	0.862	0.900	0.643
**RO**	0.905	0.907	0.925	0.638

As shown in Table 6, all HTMT values between the constructs ranged from 0.043 to 0.564 and were below 0.85. The highest value was observed between perceived effort–reward imbalance and perceived AI threat (HTMT = 0.564), which remained well below the recommended threshold, indicating good discriminant validity among the constructs.

**Table 6:** HTMT matrix for discriminant validity assessment.

Construct	AIA	AISE	EHAI	ERI	GM	PC	PTAI
AISE	0.065						
EHAI	0.201	0.39					
ERI	0.227	0.161	0.044				
GM	0.083	0.393	0.41	0.11			
PC	0.166	0.366	0.381	0.043	0.381		
PTAI	0.298	0.265	0.144	0.564	0.138	0.334	
RO	0.398	0.124	0.361	0.404	0.248	0.159	0.489

### 4.3 Structural model assessment

Before assessing the structural model, multicollinearity among the predictor variables was examined. In general, VIF values below 3.0 indicate that serious collinearity is absent (Hair et al., 2022). As shown in Table 7, all inner VIF values for the predictor variables were low, ranging from 1.162 to 1.678 and well below the recommended threshold of 3.0. The highest VIF value was observed for the relationship in which role overload (RO) predicted perceived AI threat (PTAI; VIF = 1.678), which remained far below the critical threshold. Therefore, the predictor variables in the model did not exhibit serious multicollinearity.

**Table 7:** Assessment of collinearity among predictors based on inner VIF values.

Construct	ERI	PC	PTAI
**AIA**	1.162	1.162	1.215
**AISE**	1.238	1.238	1.332
**EHAI**	1.369	1.369	1.548
**ERI**	–	–	1.32
**GM**	1.27	1.27	1.408
**PC**	–	–	1.593
**RO**	1.278	1.278	1.678

To further evaluate the practical contribution of each predictor to the explanation of the endogenous variables in the structural model, effect sizes (*f* ^2^) were calculated. According to Cohen's (1988) criteria, f^2^ values of 0.02, 0.15, and 0.35 generally represent small, medium, and large effects, respectively. As shown in Table 8, the effect size of perceived effort–reward imbalance on perceived AI threat approached a medium effect (*f* ^2^ = 0.154), and the effect size of role overload on perceived effort–reward imbalance also approached a medium effect (*f* ^2^ = 0.146). The effect size of human–AI relationship experience on perceived controllability was 0.118, making it the most important single predictor of this endogenous variable. Most of the remaining significant paths exhibited small effects. The explanatory power of the structural model for the endogenous variables was further assessed using the coefficient of determination (*R*^2^). As suggested by Hair et al. (2022), *R*^2^ measures the proportion of variance in an endogenous variable explained by the exogenous variables, with higher values indicating stronger explanatory power. As shown in Table 9, the model had a certain level of explanatory power for all three endogenous variables. The *R*^2^ value for perceived effort–reward imbalance (ERI) was 0.194, indicating that predictors such as role overload, growth mindset, and AI self-efficacy explained 19.4% of the variance in ERI. The *R*^2^ value for perceived controllability (PC) was 0.332, indicating that human–AI relationship experience, AI anxiety, AI self-efficacy, growth mindset, and role overload explained 33.2% of the variance in perceived controllability. For the core outcome variable, perceived AI threat (PTAI), the model explained 42.9% of the variance (*R*^2^ = 0.429), indicating that the proposed model explained a substantial proportion of the variation in university students' perceived AI threat.

**Table 8:** Results of the *f* ^2^ calculations.

Construct	ERI	PC	PTAI
AIA	0.008	0.027	0.014
AISE	0.02	0.038	0.018
EHAI	0.001	0.118	0.031
ERI	–	–	0.154
GM	0.017	0.068	0
PC	–	–	0.02
RO	0.146	0.092	0.11

**Table 9:** Results for *R*^2^.

Construct	*R*-square	*R*-square adjusted
ERI	0.194	0.187
PC	0.332	0.326
PTAI	0.429	0.422

To further assess the predictive relevance of the structural model, the Stone–Geisser *Q*^2^ statistic was examined. *Q*^2^ was calculated using the blindfolding procedure and was used to assess the predictive relevance of the PLS-SEM model for the endogenous variables. A *Q*^2^ value greater than zero indicates predictive relevance (Hair et al., 2022). As shown in Table 10, the Q^2^ values for perceived effort–reward imbalance, perceived controllability, and perceived AI threat were 0.132, 0.234, and 0.271, respectively, all of which were greater than zero, indicating that the model had predictive relevance for all three endogenous constructs.

**Table 10:** Stone-GEISSER *Q*^2^ predictive relevance results.

Construct	SSO	SSE	Q^2^ (=1-SSE/SSO)
ERI	2,740	2,378.81	0.132
PC	5,480	4,200.299	0.234
PTAI	2,740	1,997.537	0.271

As shown in Table 11, all five antecedent paths to perceived controllability were significant. Human–AI relationship experience (β = 0.329, *p* < 0.001), AI self-efficacy (β = 0.177, *p* < 0.001), and growth mindset (β = 0.240, *p* < 0.001) positively predicted perceived controllability, whereas AI anxiety (β = −0.145, *p* = 0.001) and role overload (β = −0.280, *p* < 0.001) negatively predicted perceived controllability.

**Table 11:** Results of direct effects in the structural model.

Path	β (Original sample)	Standard error	*t*-value	*p*-value
AIA → ERI	0.088	0.046	1.898	0.058
AIA → PC	−0.145	0.042	3.478	0.001
AIA → PTAI	0.097	0.039	2.523	0.012
AISE → ERI	−0.14	0.045	3.12	0.002
AISE → PC	0.177	0.041	4.359	< 0.001
AISE → PTAI	−0.118	0.04	2.933	0.003
EHAI → ERI	−0.025	0.046	0.534	0.593
EHAI → PC	0.329	0.04	8.164	< 0.001
EHAI → PTAI	−0.165	0.044	3.733	< 0.001
ERI → PTAI	0.341	0.044	7.779	< 0.001
GM → ERI	−0.133	0.046	2.923	0.003
GM → PC	0.24	0.037	6.481	< 0.001
GM → PTAI	−0.014	0.041	0.342	0.732
PC → PTAI	−0.135	0.046	2.931	0.003
RO → ERI	0.387	0.048	8.069	< 0.001
RO → PC	−0.28	0.043	6.52	< 0.001
RO → PTAI	0.324	0.052	6.228	< 0.001

β represents standardized path coefficients. Significance levels were determined using a bootstrapping procedure with 5,000 resamples.

For perceived effort–reward imbalance, role overload had a significant positive effect (β = 0.387, *p* < 0.001), whereas AI self-efficacy (β = −0.140, *p* = 0.002) and growth mindset (β = −0.133, *p* = 0.003) had significant negative effects. Thus, H3b, H4b, and H5b were supported. The effects of AI anxiety (β = 0.088, *p* = 0.058) and human–AI relationship experience (β = −0.025, *p* = 0.593) did not reach statistical significance;

For perceived AI threat, perceived effort–reward imbalance significantly and positively predicted threat perception (β = 0.341, *p* < 0.001), whereas perceived controllability significantly and negatively predicted threat perception (β = −0.135, *p* = 0.003). Thus, H6 and H7 were supported. Human–AI relationship experience (β = −0.165, *p* < 0.001) and AI self-efficacy (β = −0.118, *p* = 0.003) had significant negative direct effects, whereas AI anxiety (β = 0.097, *p* = 0.012) and role overload (β = 0.324, *p* < 0.001) had significant positive direct effects. The direct effect of growth mindset was not significant (β = −0.014, *p* = 0.732);

As shown in Table 12, perceived controllability significantly mediated the relationships between all five antecedent variables and perceived AI threat. Human–AI relationship experience, AI self-efficacy, and growth mindset reduced perceived threat by increasing perceived controllability, whereas AI anxiety and role overload increased perceived threat by reducing perceived controllability. With the exception of growth mindset, the other four paths exhibited partial mediation. Because the direct effect of growth mindset was not significant, it exhibited indirect-only mediation.

**Table 12:** Bootstrapped mediation effects of perceived controllability and perceived effort–reward imbalance.

Indirect Path	β	SE	*t*-value	*p*-value	95% CI	Mediation type
AIA → PC → PTAI	0.02	0.01	2.056	0.04	[0.004, 0.041]	Partial mediation
AISE → PC → PTAI	−0.024	0.01	2.423	0.015	[−0.045, −0.007]	Partial mediation
AIA → ERI → PTAI	0.03	0.017	1.767	0.077	[−0.002, 0.065]	No mediation
EHAI → PC → PTAI	−0.045	0.015	2.907	0.004	[−0.075, −0.015]	Partial mediation
AISE → ERI → PTAI	−0.048	0.017	2.827	0.005	[−0.082, −0.017]	Partial mediation
EHAI → ERI → PTAI	−0.008	0.016	0.535	0.592	[−0.040, 0.023]	No mediation
GM → PC → PTAI	−0.033	0.012	2.686	0.007	[−0.058, −0.010]	Indirect-only mediation
GM → ERI → PTAI	−0.045	0.016	2.851	0.004	[−0.077, −0.015]	Indirect-only mediation
RO → PC → PTAI	0.038	0.013	2.862	0.004	[0.012,0.064]	Partial mediation
RO → ERI → PTAI	0.132	0.021	6.411	< 0.001	[0.093,0.173]	Partial mediation

CI, confidence interval.

Partial mediation indicates that both direct and indirect effects were significant.

Indirect-only mediation indicates that the indirect effect was significant while the direct effect was not significant.

No mediation indicates that the bootstrap confidence interval included zero.

Perceived effort–reward imbalance significantly mediated the relationships of AI self-efficacy (β = −0.048, *p* = 0.005), growth mindset (β = −0.045, *p* = 0.004), and role overload (β = 0.132, *p* < 0.001) with perceived AI threat. Among these effects, the indirect effect of role overload through perceived effort–reward imbalance was the largest. The confidence intervals for the corresponding indirect effects of AI anxiety and human–AI relationship experience included zero.

**Figure 2:**
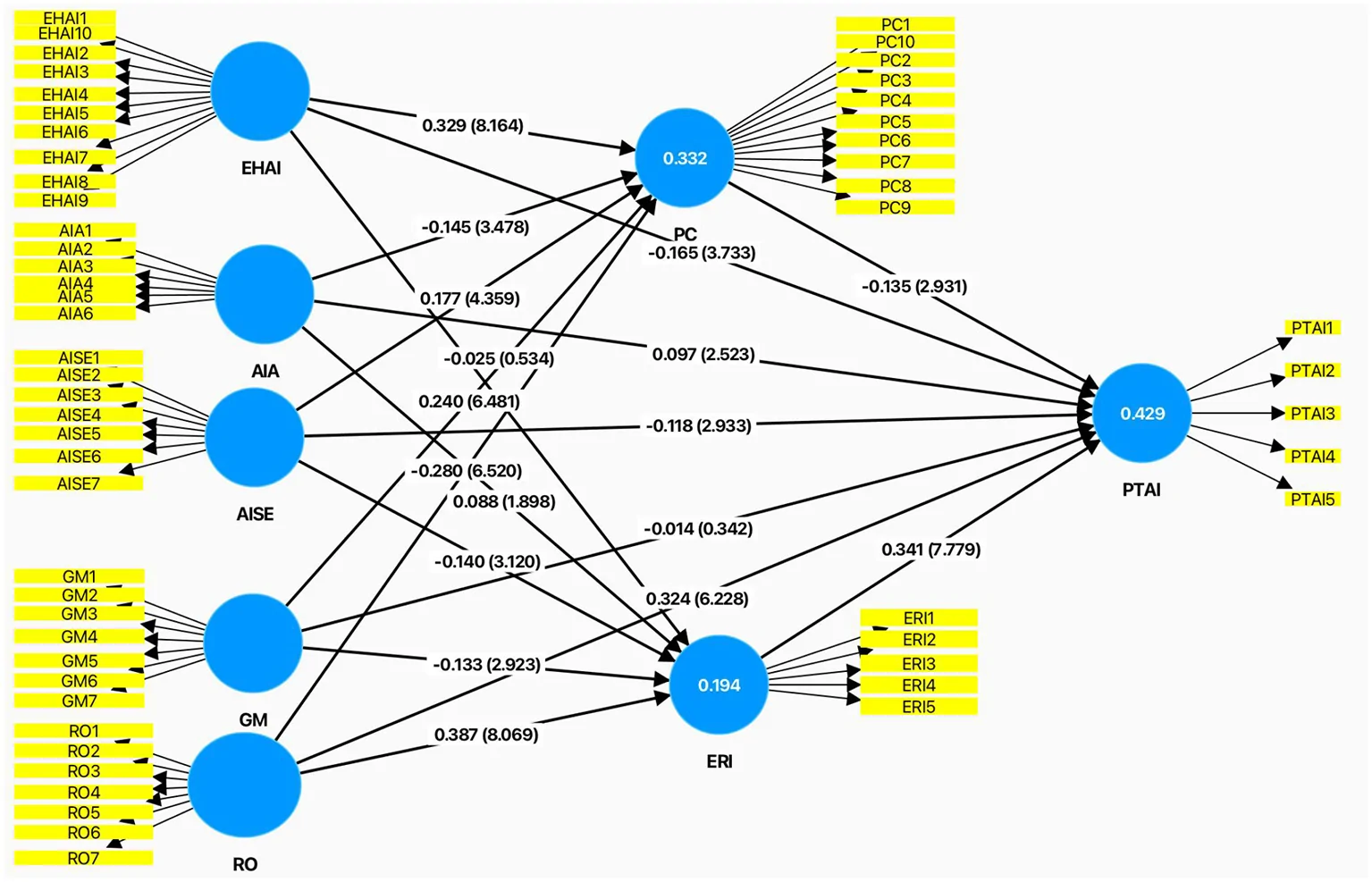
Research model and hypothesis testing results.

The total effects of each predictor on perceived AI threat were further analyzed. As shown in Table 13 and Figure 2, the total effects of all five antecedent variables were significant. Role overload had the largest total effect (β = 0.494, *p* < 0.001), followed by human–AI relationship experience (β = −0.218, *p* < 0.001), AI self-efficacy (β = −0.190, *p* < 0.001), AI anxiety (β = 0.147, *p* < 0.001), and growth mindset (β = −0.092, *p* = 0.023). Although growth mindset had no significant direct effect, its two significant indirect effects resulted in a significant total effect.

**Table 13:** Total effects of antecedent variables on perceived AI threat.

Path	β	SE	*t*-value	*p-*value
AIA → PTAI	0.147	0.04	3.661	< 0.001
AISE → PTAI	−0.19	0.038	4.988	< 0.001
EHAI → PTAI	−0.218	0.042	5.132	< 0.001
GM → PTAI	−0.092	0.041	2.271	0.023
RO → PTAI	0.494	0.046	10.75	< 0.001

### 4.4 Alternative model comparison

As shown in Table 14, to further validate the proposed dual-appraisal framework, this study constructed two competing models and compared them with the original model. The first competing model retained only the control-appraisal pathway, whereas the second retained only the effort–reward appraisal pathway. As shown in Table 14, the dual-appraisal model had the greatest explanatory power (*R*^2^ = 0.429), exceeding that of the control-appraisal-only model (*R*^2^ = 0.342) and the effort–reward-appraisal-only model (*R*^2^ = 0.419). Although all three models exhibited good model fit (all SRMR values were below 0.08), the dual-appraisal model explained more variance in perceived AI threat. This result further supports the dual-appraisal framework proposed in this study, indicating that the dual-appraisal model provided modestly greater explanatory power and supported the complementary contribution of the two appraisal pathways.

**Table 14:** Alternative model comparison.

Model	Description	PTAI *R*^2^	Adjusted *R*^2^	SRMR	NFI
Model 1	Control-appraisal-only model	0.342	0.335	0.033	0.921
Model 2	Effort–reward-appraisal-only model	0.419	0.412	0.035	0.918
Model 3	Proposed dual-appraisal model	0.429	0.422	0.041	0.91

### 4.5 Robustness analysis

To examine whether the demographic characteristics of the sample affected the core conclusions, female status, fourth-year undergraduate status, management major, and regular undergraduate institution status were dummy-coded as control variables and specified as predictors of perceived AI threat. As shown in Tables 15–17, the control variables predicted only perceived AI threat; therefore, the R^2^ values for perceived controllability and perceived effort–reward imbalance remained unchanged.

**Table 15:** Comparison of structural paths.

Path	Baseline β	Baseline *p*	Controlled β	Controlled *p*	Δβ	Interpretation
AIA → ERI	0.088	0.058	0.088	0.058	0	Unchanged
AIA → PC	−0.145	0.001	−0.145	0.001	0	Unchanged
AIA → PTAI	0.097	0.012	0.096	0.012	−0.001	Stable
AISE → ERI	−0.140	0.002	−0.140	0.002	0	Unchanged
AISE → PC	0.177	< 0.001	0.177	< 0.001	0	Unchanged
AISE → PTAI	−0.118	0.003	−0.084	0.048	0.034	Weaker, but remains significant
EHAI → ERI	−0.025	0.593	−0.025	0.593	0	Unchanged
EHAI → PC	0.329	< 0.001	0.329	< 0.001	0	Unchanged
EHAI → PTAI	−0.165	< 0.001	−0.158	< 0.001	0.007	Stable
GM → ERI	−0.133	0.003	−0.133	0.003	0	Unchanged
GM → PC	0.24	< 0.001	0.24	< 0.001	0	Unchanged
GM → PTAI	−0.014	0.732	−0.017	0.667	−0.003	Consistently non-significant
RO → ERI	0.387	< 0.001	0.387	< 0.001	0	Unchanged
RO → PC	−0.280	< 0.001	−0.280	< 0.001	0	Unchanged
RO → PTAI	0.324	< 0.001	0.32	< 0.001	−0.004	Stable
ERI → PTAI	0.341	< 0.001	0.328	< 0.001	−0.013	Stable
PC → PTAI	−0.135	0.003	−0.115	0.01	0.02	Weaker, but remains significant

β, standardized path coefficient; Δβ, controlled coefficient minus baseline coefficient.

Bootstrap samples = 5,000.

SmartPLS values of *p* = 0.000 are reported as *p* < 0.001.

**Table 16:** Demographic control effects in the controlled model.

Control path	β	SE	*t*	*p*
Female status → PTAI	0.286	0.07	4.072	< 0.001
Fourth-year undergraduate status → PTAI	0.046	0.067	0.688	0.491
Management major → PTAI	−0.133	0.072	1.846	0.065
Regular undergraduate institution → PTAI	−0.061	0.064	0.94	0.347

**Table 17:** Explained variance.

Endogenous construct	Baseline *R*^2^	Controlled *R*^2^	Baseline adjusted *R*^2^	Controlled adjusted *R*^2^
ERI	0.194	0.194	0.187	0.187
PC	0.332	0.332	0.326	0.326
PTAI	0.429	0.452	0.422	0.441

After the control variables were included, the *R*^2^ for perceived AI threat increased from 0.429 to 0.452, and the adjusted R^2^ increased from 0.422 to 0.441. Female status significantly and positively predicted perceived AI threat (β = 0.286, *p* < 0.001), whereas the other three control variables were not significant. The directions of all core paths remained unchanged; paths that were originally significant remained significant, and the originally non-significant direct effect of growth mindset remained non-significant. The coefficient for perceived effort–reward imbalance predicting perceived threat decreased slightly from 0.341 to 0.328, and the coefficient for perceived controllability changed from −0.135 to −0.115. The direct effect of AI self-efficacy weakened but remained significant (β = −0.084, *p* = 0.048). Therefore, the main conclusions demonstrated good robustness.

## 5 Discussion

### 5.1 Main findings

This study developed and validated an integrated model comprising five antecedent variables, two parallel mediators, and perceived AI threat. The findings indicate that perceived controllability and perceived effort–reward imbalance play mediating roles in opposite directions in the formation of perceived AI threat. Higher perceived controllability reduces students' perceived AI threat, whereas higher perceived effort–reward imbalance intensifies such threat perceptions. Comparatively, perceived controllability functions as a more general mediating mechanism, significantly transmitting the effects of human–AI relationship experience, AI anxiety, AI self-efficacy, growth mindset, and role overload. By contrast, the mediating role of perceived effort–reward imbalance is more selective and primarily transmits the effects of AI self-efficacy, growth mindset, and role overload. Role overload exhibited the strongest total effect. Its influence operated not only by reducing perceived controllability and increasing perceived effort–reward imbalance but also through a direct effect on perceived AI threat. Growth mindset did not produce a significant direct effect; however, it generated protective indirect effects by increasing perceived controllability and reducing perceived effort–reward imbalance. Overall, these findings indicate that whether students perceive AI as a threat to their learning and future development depends simultaneously on their judgments of “whether they can cope with the change” and “whether continued investment is worthwhile.”

### 5.2 The differential mediating roles of perceived controllability and perceived effort–reward imbalance

Although perceived controllability and perceived effort–reward imbalance both represent cognitive appraisals formed by students when confronting AI-related change, they reflect different levels of judgment. Perceived controllability represents a broader coping appraisal and concerns whether students believe that they possess the capabilities and resources required to understand, manage, and adapt to AI-related change. Control–value theory proposes that when individuals perceive important events as difficult to control, they are more likely to experience anxiety, helplessness, and threat appraisals (Pekrun, 2006). By contrast, perceived effort–reward imbalance represents a more specific cost–benefit appraisal and concerns whether the time, energy, and learning costs invested in adapting to AI can be translated into corresponding improvements in capability, academic performance, and career opportunities (Siegrist, 1996). Thus, perceived controllability addresses the question “Can I cope with this?,” whereas perceived effort–reward imbalance addresses the question “Is this investment worthwhile?.”

This distinction may explain why human–AI relationship experience and AI anxiety primarily operate through perceived controllability. Positive human–AI relationship experience helps students understand AI's capability boundaries, operating mechanisms, and predictability and reduces unfamiliarity with the technology through continued use, feedback, and strategy adjustment. Actual interaction experience may also foster “learned trust,” enabling users to judge the conditions under which AI is reliable and appropriate to depend on (Hoff and Bashir, 2015). Therefore, human–AI relationship experience primarily shapes whether students believe they can understand and manage AI, but it does not necessarily alter their judgments regarding long-term educational investment and career returns. Even when students are familiar with AI tools, they may remain uncertain about whether relevant skills will generate stable developmental returns.

AI anxiety similarly affects students' coping appraisals more directly. Anxiety may cause students to focus excessively on operational failure, inadequate capability, occupational displacement, and future uncertainty, thereby leading them to underestimate their ability to adapt to AI-related change (Wang and Wang, 2022). Under such conditions, students may be more likely to avoid practice, reduce exploration, and interpret temporary difficulties as evidence of inadequate personal capability. AI anxiety may therefore increase perceived threat by weakening perceived controllability. However, anxiety does not necessarily mean that students regard AI-related learning as lacking value. Some students with relatively high levels of anxiety may still recognize the academic and employment value of AI-related skills. In other words, anxiety primarily changes the judgment “Am I capable of doing this?” rather than consistently changing the judgment “Will doing this be worthwhile?.”

By contrast, AI self-efficacy, growth mindset, and role overload simultaneously involve capability-related resources and the perceived value of investment and can therefore influence both mediators. AI self-efficacy affects not only whether students believe that they can learn and use AI but also their expectations regarding the outcomes of learning investments (Compeau and Higgins, 1995). Growth mindset makes students more likely to view difficulties as temporary problems that can be overcome through practice and strategy adjustment, while interpreting effort as a necessary process of capability development rather than as evidence that investment is ineffective (Yeager and Dweck, 2020). Role overload operates in the opposite direction. Excessive coursework, skill requirements, and employment-preparation activities consume students' time, energy, and cognitive resources, thereby reducing their confidence in coping with AI-related change and increasing their perceptions of the costs associated with AI learning. Accordingly, the two mediators are not substitutes for one another but instead reflect, respectively, students' appraisals of their capacity to cope with AI-related change and the value of their investments in adapting to it.

### 5.3 Role overload as the most important predictor

Role overload exhibited the strongest total effect, indicating that students' perceived AI threat depends not only on the technology itself but also on their existing task demands and available resources. Role overload continuously depletes students' time, energy, and cognitive resources, making additional requirements for AI learning and capability updating more likely to be appraised as stressful and threatening. Conservation of resources theory proposes that when individuals' resources have already been depleted or are at risk of further loss, new environmental demands are more likely to elicit negative appraisals (Hobfoll, 1989). At the same time, role overload may undermine students' self-regulated learning and cognitive engagement. Because goal setting, time planning, process monitoring, and strategy adjustment all require sustained cognitive resources (Zimmerman, 2002), students experiencing high levels of overload may focus more on completing tasks as quickly as possible than on understanding and using AI effectively and in depth. They may consequently adopt short-term strategies such as surface learning, mechanical dependence, or avoidance (Fredricks et al., 2004). Role overload therefore emerged as the most important predictor because it simultaneously weakens students' resource base, their capacity for learning regulation, and their confidence in adapting to AI-related change.

### 5.4 Growth mindset produces only indirect effects

Growth mindset did not directly predict perceived AI threat but generated significant protective indirect effects by increasing perceived controllability and reducing perceived effort–reward imbalance. This finding suggests that growth mindset does not cause students to deny the potential risks of skill displacement, disciplinary competition, and career uncertainty associated with AI. Instead, it influences how they interpret difficulties and continued investment. Students with a growth mindset are more likely to regard operational difficulties, knowledge gaps, and tool updates as problems that can be gradually overcome through practice, strategy adjustment, and help seeking, thereby strengthening their sense of control over AI-related change (Yeager and Dweck, 2020). At the same time, they are more likely to view effort as a long-term investment in capability development and future adaptation rather than as evidence that their investment is ineffective and are therefore less likely to experience a mismatch between effort and reward. This process may also help students maintain learning motivation, strategic persistence, and cognitive engagement when facing pressure and failure, thereby reflecting stronger academic resilience (Martin and Marsh, 2006). Thus, growth mindset does not directly reduce students' judgments of AI-related risk; rather, it exerts a protective effect by strengthening their coping confidence and their perceptions of the value of effort.

### 5.5 Discussion of perceived AI threat

This study further extends the understanding of the relationship between students and AI. Existing technology-acceptance research has mainly focused on whether students are willing to use AI, typically emphasizing perceived usefulness, perceived ease of use, and behavioral intention (Davis, 1989). However, students may recognize the usefulness of AI and use AI tools frequently while still worrying about the devaluation of disciplinary knowledge, declining returns on learning investments, and threats to future career opportunities. This indicates that willingness to use AI and perceived AI threat are not the same issue. The present study also demonstrates that perceived AI threat cannot simply be understood as an extension of AI anxiety. Whether students perceive AI as threatening is influenced not only by negative emotions such as fear, tension, and concerns about occupational displacement (Wang and Wang, 2022), but also by whether they believe that they can manage relevant changes and whether continued investments of time and effort will generate corresponding returns. By examining perceived controllability and perceived effort–reward imbalance simultaneously, this study identifies two complementary appraisal processes—“whether one can cope” and “whether the investment is worthwhile”—and thereby provides a more comprehensive explanation of university students' threat judgments in the context of AI-related change.

### 5.6 Theoretical implications

This study makes several theoretical contributions. First, it distinguishes perceived AI threat from research on general attitudes toward technology and behavioral intention, emphasizing that threat perception is a subjective judgment connected to individuals' own opportunities, the value of their capabilities, and career development. The findings show that technology itself does not directly determine the level of threat; individuals' interaction experience, capability beliefs, emotions, and resource conditions are also important. Second, this study tested perceived controllability and perceived effort–reward imbalance as two mediators within the same model and demonstrated through specific indirect effects that two distinct cognitive judgments can simultaneously explain the effects of some antecedent variables. Perceived controllability transmitted the effects of all five antecedent variables more broadly, whereas perceived effort–reward imbalance was more sensitive to role overload, growth mindset, and AI self-efficacy. Third, this study extends the populations and contexts examined in AI education research. Existing AI-related research has largely focused on students in technical disciplines, AI users, or employees in organizations, while insufficient attention has been paid to humanities and social sciences students, a group that may face relatively high career uncertainty. By focusing on this group, the study reveals how students in non-technical disciplines understand their own capabilities, learning investments, and future development opportunities as AI rapidly enters education and employment. This perspective complements the excessive emphasis on developing technical capabilities in existing AI education research and highlights that students' psychological adaptation and developmental expectations are also important components of educational transformation in the AI era. Finally, the non-significant findings further clarify the boundaries of the mechanisms underlying perceived AI threat. The study found that growth mindset does not directly reduce perceived AI threat but operates indirectly mainly by changing students' appraisals of their own capability development and the value of their investments. Therefore, this study not only verifies important factors influencing perceived AI threat but also further clarifies the conditions and pathways through which these factors operate.

### 5.7 Practical implications

The findings have important implications for AI education practices and student development support in universities. First, higher education institutions should not focus solely on developing students' AI skills but should also attend to the psychological adaptation process students experience when facing AI-related change. This study found that perceived controllability is an important factor in reducing perceived AI threat. Universities can therefore help students develop a reasonable understanding of AI capabilities and application boundaries by providing systematic AI education, practical courses, and authentic opportunities for human–AI collaboration. Compared with merely introducing trends in AI development, increasing opportunities for students to use AI tools in practice may be more effective in increasing their sense of control and reducing threat perceptions generated by unfamiliarity and uncertainty. Second, universities need to pay attention to AI anxiety and difficulties in technological adaptation. The results showed that AI anxiety not only reduced students' perceived controllability but also directly increased perceived AI threat. Therefore, when promoting the integration of AI into teaching, universities should not emphasize only the efficiency gains offered by AI but should also attend to the sense of inadequate capability and future uncertainty that some students may experience. For example, basic AI training, interdisciplinary AI courses, and application-oriented teaching for students in non-technical disciplines may reduce the psychological barriers students face when entering AI environments. Third, this study emphasizes the importance of enhancing AI self-efficacy. AI self-efficacy not only reduced students' perceptions of AI threat but also reduced their perceived effort–reward imbalance. This means that universities should avoid limiting AI education to operational skills and should help students develop confidence that they are able to learn about and adapt to AI-related change. Particularly in the context of rapid AI development, students' future competitiveness depends not only on how much AI knowledge they currently possess but also on whether they believe they can continue learning and adjusting the structure of their capabilities. Finally, this study has implications for university AI governance and student career development guidance. Reducing students' perceived AI threat does not mean reducing discussion of the potential risks of AI; rather, it means helping students develop a more balanced and realistic understanding of AI. Universities can therefore guide students to understand the relationship between AI and future career development through career-planning education, discussions of AI ethics, and human–AI collaboration practice, enabling students to shift from passively confronting technological change to actively participating in the adaptation process.

### 5.8 Limitations and future research

Although this study further reveals the mechanisms underlying university students' perceived AI threat, several limitations remain and should be explored in future research. First, this study employed a cross-sectional questionnaire design and therefore primarily reveals associations among variables rather than permitting strict causal inference. Although the paths among the variables were proposed on the basis of the theoretical model and previous research, perceived AI threat, AI anxiety, and perceived controllability may change dynamically as students gain more experience using AI. Future research could therefore adopt longitudinal designs to examine how students' threat perceptions and psychological adaptation change as AI becomes increasingly integrated into learning and career development. Experimental research could also further test whether AI-use experience, capability training, or positive human–AI interaction experience can effectively reduce perceived threat. Second, the sample consisted mainly of humanities and social sciences students in the Chinese higher education system; therefore, the applicability of the findings should be interpreted cautiously. This group was selected mainly because, compared with students in technology-related disciplines, humanities and social sciences students may face more salient skill transformation and career uncertainty. However, students with different disciplinary backgrounds, at different educational stages, and at different stages of career development may have different AI experiences and risk judgments. For example, students in computer-related disciplines may exhibit different threat-appraisal patterns because of their greater technological familiarity, whereas graduates who have already entered the labor market may focus more closely on the actual effects of AI on career development. Future research could compare the mechanisms underlying perceived AI threat across disciplines, educational stages, and occupational groups to enhance the external validity of the findings. Third, this study treated AI as an overarching category of technology and did not further distinguish the differentiated effects that different AI applications may have. Students' threat perceptions may differ across generative AI tools, AI recruitment systems, automated grading systems, intelligent recommendation systems, and adaptive learning systems. For example, generative AI may generate greater concerns about learning capabilities and the value of knowledge, whereas AI recruitment systems may generate greater concerns about employment fairness and career opportunities. Future research could therefore distinguish specific AI application contexts and construct more context-specific models of perceived AI threat. Fourth, this study relied primarily on participants' self-reported data to measure the variables. Although reliability and validity assessments and tests of common method bias were conducted to improve measurement reliability, the effects of social desirability and subjective cognitive bias cannot be completely ruled out. Future research could combine multiple data sources, such as actual AI-use records, learning behavior data, interview data, or performance on experimental tasks, to develop a more comprehensive understanding of how students form and adjust their threat judgments regarding AI. Future research could also further explore whether actual AI-use behavior changes the relationship between perceived controllability and effort–reward appraisal. Fifth, the measurement instruments were shortened, extended, and contextually adapted to varying degrees. In particular, two items were added to perceived controllability on the basis of the original perceived constraints dimension, two items were added to the original five-item role overload measure, and a five-item perceived effort–reward imbalance scale was newly developed for this study on the basis of ERI theory. Although the outer loadings, internal consistency, convergent validity, and discriminant validity of these constructs met the recommended criteria in the current sample, good performance in a single sample cannot substitute for complete scale development and cross-sample validation. Future research should use independent samples to conduct exploratory and confirmatory factor analyses and examine structural stability, test–retest reliability, criterion-related validity, measurement invariance across groups, and incremental validity. In particular, future research should compare the overall perceived effort–reward imbalance scale with the traditional ERI dimensions and ERI ratio. Finally, this study focused on individual-level psychological and contextual factors and did not include more macro-level variables such as university AI policies, course provision, teacher support, regional employment structures, and media narratives. Subsequent research could use multilevel models to integrate individual resources with educational organizations, labor markets, and social information environments.

## 6 Conclusion

This study examined the factors influencing perceived AI threat among Chinese university students majoring in the humanities and social sciences and the mediating mechanisms involved. The results showed that perceived controllability significantly reduced perceived AI threat, whereas perceived effort–reward imbalance significantly increased perceived AI threat. Perceived controllability mediated the effects of human–AI relationship experience, AI anxiety, AI self-efficacy, growth mindset, and role overload. Perceived effort–reward imbalance mediated the effects of AI self-efficacy, growth mindset, and role overload. Role overload was the risk factor with the largest overall effect, whereas growth mindset operated protectively mainly through indirect pathways. The findings indicate that helping university students adapt to the AI era requires not only enhancing their actual capabilities and human–AI interaction experience but also reducing their sense of external constraints and role overload and enabling them to recognize realistic connections between learning investments and future development.

## Data Availability

The raw data supporting the conclusions of this article will be made available by the authors, without undue reservation.
