# Supplementary material for: Mechanisms underlying university students' perceived AI threat: the mediating roles of perceived controllability and perceived effort–reward imbalance
Source: Front Psychol. 2026 Aug 25;17:1875419. doi: 10.3389/fpsyg.2026.1875419 (PMC13575752; doi:10.3389/fpsyg.2026.1875419)
Supplement: Supplementary file 1 [file Data_Sheet_1.docx]

Appendix 1. Sources, Measurement Content, and Adapted Versions of the Research Instruments

| **Construct** | **Source Scale** | **Original Scale** | **Number of Items Used in This Study** | **Measurement Content in This Study** | **Main Adaptation Procedures** |
| --- | --- | --- | --- | --- | --- |
| AI Anxiety | Adapted and shortened from Yang and Sundar (2025) | The original scale consisted of seven items. It measured a relatively stable tendency toward tension, worry, unease, and vigilance arising from the potential negative effects of artificial intelligence. | 6 | Students’ tendencies toward tension, worry, unease, and anxiety when encountering, using, or thinking about artificial intelligence and its potential effects. | Six items highly relevant to university students’ use of artificial intelligence were selected from the original seven-item measure of AI trait anxiety, and one item that was less appropriate for the context of the present study or substantially overlapped with the content of perceived AI threat was removed. The general artificial intelligence context in the items was adapted to situations in which university students encounter, use, and think about artificial intelligence during learning, personal development, and future career preparation. The core emotional meanings of tension, worry, unease, and anxiety were retained during the adaptation. |
| Perceived Controllability | Adapted and extended from Lachman and Weaver (1998a, 1998b) | The original sense of control scale contains 12 items comprising two related dimensions: personal mastery (4 items) and perceived constraints (8 items). Personal mastery reflects individuals’ beliefs in their ability to achieve goals and influence outcomes; perceived constraints reflect the extent to which individuals believe that goal attainment is restricted by external obstacles or uncontrollable factors. | 10 | The extent to which students perceive fewer restrictions from external obstacles, environmental conditions, and other uncontrollable factors when adapting to AI-related learning environments and future career changes. | Eight items were selected from the perceived constraints dimension. The general life context in the original items was adapted to AI-related learning environments and future career changes, and two contextualized items were added based on the theoretical definition of perceived constraints to reflect the external and contextual constraints that students may perceive when adapting to AI-related educational and career changes. All items were worded in the direction of constraints and were reverse-coded before data analysis. Therefore, higher scores indicate that students perceive fewer AI-related constraints, that is, stronger AI-related perceived controllability. |
| AI Self-Efficacy | Adapted and shortened from Hong (2022) | The original scale contains 10 items in one dimension and assesses whether individuals believe that they can still use artificial intelligence technology to complete the required tasks under different conditions, such as in the absence of guidance, without prior experience, or with only limited assistance. | 7 | Students’ beliefs in their ability to learn, independently use, and effectively apply artificial intelligence tools to complete learning and related tasks. | Seven items that directly reflect individuals’ ability to use artificial intelligence were selected from the original 10-item measure of AI self-efficacy. To reduce content overlap between AI self-efficacy and social support or external facilitating conditions, three items that mainly depended on direct assistance, guidance, or demonstration from others were removed. The retained items mainly reflect whether students still believe that they can learn and use artificial intelligence to complete tasks when they lack in-person guidance, have no experience using similar tools, can refer only to relevant materials, or receive limited system assistance. The general artificial intelligence technology use context in the original items was adapted to situations in which university students use generative artificial intelligence to complete learning, information-processing, and related tasks. |
| Growth Mindset | Adapted and shortened from Sigmundsson and Haga (2024) | The original scale contains one dimension and eight items and assesses the extent to which individuals believe that their skills, knowledge, and abilities can be continuously developed through effort, practice, learning, and accepting challenges. | 7 | Students’ belief that their abilities can be continuously improved through effort and learning. | Items related to ability development, effort, and the learning process were retained. Based on the relevance of the items to the university student learning context and their semantic appropriateness, one item that was less well matched to the context of the present study was removed. Because growth mindset is a general personality disposition, the items were not fully contextualized to artificial intelligence; only necessary adjustments were made to individual expressions. |
| Role Overload | Adapted and extended from Peterson et al. (1995) | The original scale contains 13 items covering role overload (5 items), role conflict (3 items), and role ambiguity (5 items). The scale was primarily designed for workplace and organizational settings and assesses whether role demands exceed the resources available to an individual. | 7 | Whether students perceive that the time, energy, or personal resources required by their learning tasks, ability-development demands, and role responsibilities exceed what is actually available to them. | The five items from the original role overload dimension were retained, and the original workplace and organizational responsibility contexts were adapted to university students’ learning tasks, ability-development demands, and student-role responsibilities. On this basis, two contextualized items were added according to the theoretical definition of role overload, including a reverse-worded item. |
| Perceived Effort–Reward Imbalance (ERI) | Developed for the present study based on the effort–reward imbalance framework of Siegrist (1996) | Effort–reward imbalance theory conceptualizes work stress as a nonreciprocal state between high effort and low reward and typically measures effort, reward, and overcommitment separately. Effort and reward are the two core components that form an appraisal of imbalance. | 5 | Students’ perception of a mismatch between their investment in learning and personal development and the corresponding rewards in academic achievement, growth and progress, or future career development. | The present study did not directly adopt the dimensional structure or the ERI ratio of the traditional ERI questionnaire. Instead, based on the core definition of nonreciprocity between investment and return in ERI theory, five items were developed in the context of university students adapting to AI-related learning and career changes to measure students’ overall subjective evaluation of the degree of mismatch between AI-related effort investment and expected academic and career returns. Therefore, this instrument is a theory-driven, contextually developed scale. |
| Perceived AI Threat | Adapted from Schepman and Rodway (2020), with supplementary structural evidence from Țîru et al. (2025) | The GAAIS developed by Schepman and Rodway ultimately contains 20 items across two dimensions: 12 items on positive attitudes toward artificial intelligence and 8 items on negative attitudes toward artificial intelligence. Țîru et al. subsequently conducted an exploratory factor analysis of the adapted eight negative-attitude items and identified a five-item Perceived AI Threat factor among the relevant negative-attitude items. | 5 | The extent to which students perceive that artificial intelligence may threaten their future employment opportunities, personal development, and career development. | Item meanings most closely related to danger, future harm, loss of control, and overall threat were selected from the negative-attitude content of the GAAIS, and the general artificial intelligence wording was contextualized to university students’ future employment and personal development. The subsequent study by Țîru et al. provided supplementary support for treating the five conceptually similar items as a unidimensional measure of perceived AI threat. Therefore, the present study measures perceived AI threat rather than general negative attitudes toward artificial intelligence. |
| Human–AI Relationship Experience | Adapted and shortened from Christoforakos et al. (2021) | The original scale contains 17 items in one dimension and measures users’ sense of social connectedness with a chatbot, regarding it as an important manifestation of the human–technology relationship. | 10 | Students’ experiences of smooth communication, mutual understanding, interaction familiarity, and positive relational connection formed through ongoing interactions with artificial intelligence. | The core content concerning communication, understanding, familiarity, and relationship in the original measure was retained. The specific Replika chatbot context was adapted to situations in which university students use generative artificial intelligence for learning activities, while items closely related to social companionship, private emotional communication, or specific chatbot functions were removed. After screening for content relevance, semantic clarity, and item redundancy, 10 items were retained. |

Note. All items in the present study were rated on a 7-point Likert scale (1 = "strongly disagree" and 7 = "strongly agree"). The reliability and validity of all adapted measurement instruments were reassessed using the current sample.
