# Supplementary material for: Mechanisms underlying university students' perceived AI threat: the mediating roles of perceived controllability and perceived effort–reward imbalance
Source: Front Psychol. 2026 Aug 25;17:1875419. doi: 10.3389/fpsyg.2026.1875419 (PMC13575752; doi:10.3389/fpsyg.2026.1875419)
Supplement: Supplementary file 2 [file Supplementary_file_1.docx]

**Participant Information Sheet and Informed Consent**

Dear Participant,

Thank you for your interest in this study.

This research aims to investigate university students' perceptions and experiences of Artificial Intelligence (AI), as well as their views on AI in relation to learning, personal development, and future career prospects. Specifically, the study seeks to explore the factors influencing university students' perceived AI threats in the context of rapid AI development.

This survey is conducted solely for academic research purposes. It is completely voluntary and has no commercial purpose. Please read the following information carefully before deciding whether to participate.

**1. Eligibility**

This study is intended for university students who meet all of the following criteria:

- Currently enrolled as an undergraduate or postgraduate student at a university in China;
- Majoring in the humanities or social sciences (e.g., Literature, History, Philosophy, Law, Economics, Management, Education, or Arts);
- Have experience using, learning about, or interacting with AI technologies or tools (e.g., ChatGPT, DeepSeek, Kimi, ERNIE Bot, Doubao, Microsoft Copilot, etc.).

If you do not meet these criteria, please do not continue with the questionnaire. We sincerely appreciate your interest.

**2. Voluntary Participation**

Your participation in this study is entirely voluntary.

You have the right to:

- Decide freely whether or not to participate;
- Withdraw from the survey at any time without providing a reason;
- Skip any question that you do not wish to answer.

Choosing not to participate or withdrawing from the study will not result in any negative consequences.

**3. Study Procedures**

The questionnaire takes approximately 10–15 minutes to complete.

There are no right or wrong answers. Please respond according to your genuine opinions and experiences.

**4. Risks and Benefits**

This study is a social science survey and does not involve any medical intervention or physical experiment. Therefore, no foreseeable physical risks are associated with participation.

Some questions relate to artificial intelligence, learning experiences, and future career development. If you feel uncomfortable while answering any question, you may discontinue the survey at any time.

Although you will not receive any direct financial benefit from participating, your responses will contribute to academic research on artificial intelligence and higher education.

**5. Privacy and Confidentiality**

This survey is conducted anonymously.

No personally identifiable information, such as your name, student ID number, national identification number, telephone number, or home address, will be collected.

All collected data will be used solely for academic research purposes and will be kept strictly confidential.

Research findings may be published in academic journals, conference proceedings, or research reports. However, only aggregated statistical results will be reported, and no information that could identify individual participants will be disclosed.

**6. Information for Participants Under the Age of 18**

Although this study primarily targets university students, a small number of university students may be under the age of 18.

If you are under 18 years old, please participate only if:

- Your parent or legal guardian has been informed about this study;
- Your parent or legal guardian agrees to your participation;
- You voluntarily agree to participate in this research.

If you have not obtained your parent or legal guardian's permission, please do not continue with this questionnaire.

**7. Contact Information**

If you have any questions regarding this study, please contact the researcher:

Email: [yuetongliang001@gmail.com](mailto:yuetongliang001@gmail.com)

**Informed Consent**

Please read the following statements carefully before proceeding.

☐ I have read and understood the information provided above.

☐ I understand that my participation is entirely voluntary and that I may withdraw at any time without penalty.

☐ I understand that my responses will remain anonymous and will be used solely for academic research purposes.

☐ If I am under 18 years of age, I confirm that my parent or legal guardian has been informed of and agrees to my participation in this study.

☐ I voluntarily agree to participate in this research.

○ Yes, I agree to participate in this study and wish to continue with the questionnaire.

○ No, I do not agree to participate. (End of Survey)

**Part II. Demographic Information**

Please select the option that best describes your current situation.

1. Gender

○ Male

○ Female

2. Age

○ Under 18

○ 18–25

○ 26–30

○ 31–40

3. Current Academic Level

○ Third-year Undergraduate

○ Fourth-year Undergraduate

○ Master's Year 2

○ Master's Year 3

○ Other (Please specify): __________

4. Major

○ Literature

○ History

○ Philosophy

○ Law

○ Economics

○ Management

○ Education

○ Arts

○ Other (Please specify): __________

5. University Type

○ Project 985 University

○ Project 211 University

○ Double First-Class University

○ Regular Undergraduate University

○ Vocational or Technical College

6. Have you ever used AI tools to support your learning or daily life?

Examples include ChatGPT, DeepSeek, Kimi, ERNIE Bot, Doubao, Microsoft Copilot, Tongyi Qianwen, and other AI-powered applications.

○ Yes

○ No

If your answer is "No", please terminate the survey.

7. Is your major classified as a Humanities or Social Sciences discipline?

Examples include Literature, History, Philosophy, Law, Economics, Management, Education, and Arts.

○ Yes

○ No (End of Survey)
